# Birth sex ratio in Western Sydney during the COVID-19 pandemic: associations by maternal country of birth

**DOI:** 10.1186/s12978-026-02304-1

**Published:** 2026-04-24

**Authors:** Lieu Thi Thuy Trinh, Hassan Assareh, Michael Piza, Veth Guevara, Sabrina Naz, Kylie Smythe, Seng Chai Chua, Kathy Eljiz

**Affiliations:** 1 Present Address: Western Sydney Local Health District, Sydney, Australia; 2 New South Wales Department of Health, Sydney, Australia; 3 University of Sydney, Sydney, Australia; 4 University of New South Wales, Sydney, Australia

**Keywords:** Birth sex ratio, COVID-19 pandemic, Immigrant, Multi-ethnicity

## Abstract

**Background:**

The sex ratio at birth (SRB)—the proportion of male to female births—is a vital demographic indicator with implications for societal stability. SRB can be influenced by a range of factors including socio-economic conditions, parental health, cultural preferences, with acute population-level stress recognised as a highly significant influence. This study examined temporal changes in SRB during the COVID-19 pandemic and associated maternal factors in a large, multicultural population.

**Methods:**

Birth data from women residing in the Western Sydney Local Health District (WSLHD) between 2016 and 2023 were analysed using multivariable logistic regression and time series analysis. Analyses adjusted for maternal demographic, socio-economic, health, and reproductive factors.

**Results:**

Of 119,756 singleton births, 61,832 were male, resulting in a sex ratio at birth (SRB) of 0.516 (106.7 males per 100 females). No significant overall change in SRB was observed during the early post-pandemic period. The SRB during the 3–5 months following the onset of the COVID-19 pandemic increased only among births to Chinese mothers, as indicated in both time series analyses and logistic regression (SRB = 0.621 vs. 0.519; adjusted odds ratio [aOR] = 1.557; 95% CI: 1.194–2.030 compared to other periods). Other factors associated with an increased SRB included having an Indian mother (SRB = 0.524; adjusted odds ratio [aOR] = 1.038; 95% CI: 1.001–1.076), compared to having a non-Indigenous Australian mother (SRB = 0.516). Higher SRBs were also observed among mothers with one previous pregnancy (SRB = 0.519 vs. 0.513; aOR = 1.031; 95% CI: 1.004–1.059) or two previous pregnancies (SRB = 0.522; aOR = 1.050; 95% CI: 1.012–1.090), compared to mothers with no previous pregnancies. Conversely, mothers with hepatitis B had a lower SRB than those without the condition (SRB = 0.483 vs. 0.517; aOR = 0.858; 95% CI: 0.755–0.976).

**Conclusions:**

The COVID-19 pandemic was not associated with an immediate aggregate shift in SRB in WSLHD. However, subgroup-specific variation by maternal country of birth was observed, highlighting the importance of maternal background in shaping SRB patterns. These findings underscore the need for culturally informed public health approaches in diverse populations during major societal disruptions.

**Supplementary Information:**

The online version contains supplementary material available at 10.1186/s12978-026-02304-1.

## Plain Language Summary

This study examined whether the COVID-19 pandemic was associated with changes in the number of boys born compared to girls in Western Sydney, Australia, and whether this differed across maternal groups.

Overall, there was no clear change in the balance between boys and girls during the early pandemic period. However, among mothers from China, a higher proportion of boys was observed a few months after the pandemic began. Mothers born in India had the highest proportion of boys throughout the study period, but no distinct change related to the early pandemic was identified for this group.

The study also found that women who had more previous pregnancies were slightly more likely to have boys, while mothers with hepatitis B were less likely to have boys. Among Chinese mothers, those living in wealthier areas were less likely to have boys compared to those in less wealthy areas. Older Australian mothers and those who were underweight were also less likely to have boys.

These findings suggest that changes in the sex ratio during the pandemic differed across cultural and social groups. Supporting families in culturally appropriate ways may help maintain a balanced number of boys and girls during future societal disruptions.

**Supplementary Information:**

The online version contains supplementary material available at 10.1186/s12978-026-02304-1.

## Introduction

The sex ratio at birth (SRB) is defined as the number of male births per total live births [1]. The global average SRB is 0.512, meaning there are approximately 105 males for every 100 females [2]. A slightly higher number of male births compared to female births is, among other evolution theories, thought to result from the single X chromosome in male foetuses, which makes them more susceptible to genetic imbalances. This, in turn, contributes to slower fetal maturation and greater vulnerability to illnesses during early development. Furthermore, male infants are generally more prone to various health conditions and diseases after birth [3, 4].

A balanced sex ratio is crucial for various aspects of society, including population growth [5], family lineage, the job market [6], economic stability [7], societal security [8], and violence against women [9, 10].

A range of factors are associated with a higher SRB, including younger [11] or older maternal age [12], higher education [11], higher parity, maternal country of birth (COB) [13], higher maternal pre-pregnancy weight or lower weight gain during pregnancy [14], greater maternal height [15], maternal hepatitis B positivity [16], and exposure to air pollution [12]. Culture also plays an important role in SRB. In countries like China and India, males are favoured over females [17, 18]. Practices such as sex-selective abortion and in vitro fertilization (IVF) have led to skewed SRBs [17, 18].

Acute population-level stress has been consistently examined as a factor associated with the SRB [19]. Stressful events such as disasters, terrorism, or economic collapse have been associated with changes in SRB. Research suggests that 3–5 months after such events, there may be a decline in male births due to higher levels of maternal stress-induced adrenal androgens, which are associated with a higher risk of miscarriage [20, 21, 22]. Moreover, maternal stress has been associated with reduced male foetal survival, resulting in a reduced SRB about nine months later [23].

The COVID-19 pandemic (the pandemic) introduced multiple stressors within countries, including disruption to healthcare access, social isolation, employment insecurity, and changes in health-seeking behaviour. These stressors may be associated with SRB through biological pathways related to maternal stress responses; however, the magnitude and direction of these associations have varied across settings.

The pandemic has been associated with changes in SRB in some countries, although findings have been inconsistent. In England and Wales, the SRB decreased three months after the onset of the pandemic but increased at nine months [1]. In contrast, Ireland experienced a decrease in SRB at nine months [24]. While in Iran, an increase was observed between months 10 and 13 [25]. In the United States, however, the pandemic had no significant association with SRB [11]. Notably, most studies on the pandemic’s associations with SRB have been limited to data collected up to December 2021, less than two years after the pandemic began.

In Australia, the pandemic was declared in March 2020, leading to widespread restrictions. In hospitals, healthcare professionals were reluctant to admit patients due to concerns about COVID-19 exposure [26]. The pandemic’s associations with maternal and newborn health services were significant, including reduced antenatal care [27], and increased caesarean section rates [28]. Australia’s SRB between 2016 and 2023 remained within the expected range (105.1 and 106.1 males per 100 females) [29]. Although previous research has shown that SRB varied by states, maternal COB and parity [13] but no study has examined temporal changes in SRB during the COVID-19 pandemic in Australia, particularly within a highly multicultural population.

The maternal characteristics examined in this study were selected because they reflect biological, reproductive, and social factors that may modify susceptibility to stress or baseline variation in SRB. Maternal age, parity, height, and pre-pregnancy body mass index (BMI) represent biological and reproductive factors that may influence foetal resilience under stressful conditions. Maternal hepatitis B status has previously been associated with SRB variation, while maternal country of birth (COB) and area-level socio-economic disadvantage capture cultural, social, and structural factors that may shape reproductive patterns and exposure to pandemic-related stressors.

Most previous studies examining SRB during the COVID-19 pandemic have been conducted in relatively homogeneous national populations and have not assessed variation across maternal subgroups. Maternal COB is a recognised determinant of SRB [13], suggesting that pandemic-related temporal changes may differ in multicultural settings.

Western Sydney Local Health District (WSLHD) has over 1 million residents and is one of the most populous areas in Sydney. Approximately two-thirds of mothers giving birth in WSLHD are immigrants [30, 31] compared to one third in Australia [32]. Half of these immigrant mothers are from South Asia, with the majority coming from India [30, 31]. This highly multicultural population provides an opportunity to examine whether SRB patterns during the COVID-19 pandemic varied by maternal COB. We aimed to examine changes in SRB during the COVID-19 period in WSLHD and to identify maternal factors associated with SRB, with particular attention to variation by COB.

## Methods

### Study design

This retrospective cohort study utilised routinely recorded data from all women residing within the WSLHD who gave birth at public or private hospitals. Data from the New South Wales Health Perinatal Data Collection, NSW Ministry of Health Secure Analytics for Population Health Research and Intelligence were analysed. The data include demographic details of the mothers (e.g., age, COB), medical history (e.g., hypertension, hepatitis B), and reproductive history (e.g., number of pregnancies) (Table 1). The analysis included all live singleton births with a gestational age of at least 20 weeks, a birth weight of at least 400 g, and a clearly identified sex of either boy or girl, recorded between 2016 and 2023. Multiple births were excluded from the analysis, as they could include both male and female infants. The time frame was selected to have four years prior to (2016–2019) and four years following the onset of the Pandemic (2020–2023).

**Table 1:** Characteristics of mothers with a singleton birth and logistic regression model of factors associated with sex ratio at birth in WSLHD between 2016 and 2023

	All	%	Males	SRB	aOR	95% CI	
Total	119,756	100.0	61,832	0.516			
TIME							
June-August 2020	3,819	3.2	1,994	0.522	1.032	0.925	1.151
December 2020	1,147	1.0	574	0.500	0.939	0.836	1.055
Other times	114,790	95.9	59,264	0.516	Ref.		
AGE (years)							
14–24	11,990	10.0	6,263	0.522	1.042	0.997	1.088
25–29	31,271	26.1	16,144	0.516	1.004	0.974	1.034
30–34	46,310	38.7	23,927	0.517	Ref.		
35–39	25,263	21.1	13,021	0.515	0.988	0.958	1.020
>=40	4,922	4.1	2,477	0.503	0.950	0.894	1.009
COUNTRY OF BIRTH							
Australia, non-Indigenous	44,597	37.2	23,000	0.516	Ref.		
India	19,380	16.2	10,148	0.524	1.038	1.001	1.076
China	8,828	7.4	4,603	0.521	1.040	0.991	1.093
Philippines	4,261	3.6	2,172	0.510	0.983	0.921	1.049
Pakistan	3,787	3.2	1,926	0.509	0.967	0.904	1.035
Nepal	3,185	2.7	1,635	0.513	0.997	0.925	1.076
Others	35,718	30.0	18,348	0.514	0.994	0.965	1.024
SEIFA INDEX							
Most disadvantaged	29,939	25.4	15,465	0.517	Ref.		
2nd most disadvantaged	21,822	18.5	11,421	0.523	1.026	0.990	1.064
2nd most advantaged	29,791	25.3	15,393	0.517	0.999	0.965	1.033
Most advantaged	36,200	30.7	18,522	0.512	0.982	0.950	1.015
HEIGHT							
Shortest decile	12,079	10.1	6,342	0.525	1.021	0.967	1.079
Medium (2nd-9th deciles)	96,389	80.5	49,648	0.515	Ref.		
Tallest decile	11,275	9.4	5,835	0.518	0.987	0.949	1.028
BODY MASS INDEX							
Underweight (< 18.5)	4,853	4.1	2,532	0.522	1.024	0.964	1.087
Healthy weight (18.5-<25)	58,208	48.6	29,986	0.515	Ref.		
Overweight (25-<30)	32,547	27.2	16,830	0.517	1.004	0.977	1.033
Obese ( > = 30)	22,916	19.1	11,822	0.516	1.001	0.970	1.034
PRE-EXISTING DIABETES							
No	118,320	98.8	61,116	0.517	Ref.		
Yes	1,436	1.2	716	0.499	0.951	0.855	1.057
HYPERTENSION							
No	118,448	98.9	61,186	0.517	Ref.		
Yes	1,308	1.1	646	0.494	0.933	0.835	1.042
HEPATITIS B							
No	118,779	99.2	61,360	0.517	Ref.		
Yes	977	0.8	472	0.483	0.858*	0.755	0.976
PREVIOUS PREGNANCY							
0	49,834	41.6	25,551	0.513	Ref.		
1	43,382	36.2	22,503	0.519	1.031*	1.004	1.059
2	16,207	13.5	8,453	0.522	1.050*	1.012	1.090
>=3	10,333	8.6	5,325	0.515	1.038	0.991	1.087

*SRB* Sex ratio at births (= males/total live babies); *p < 0.05; **p < 0.01; ***p < 0.001

### Factors associated with SRB

The SRB was defined as the proportion of males per total live births. The periods of interest were three to five months (June - August 2020) and nine months (December 2020) following the onset of the pandemic. These lag periods were selected based on previous literature linking acute population stress to early gestational male vulnerability (3–5 months) and stress-related changes in conception patterns (approximately nine months). In addition to the pandemic periods, maternal demographic, socio-economic, health, and reproductive characteristics were included as potential factors associated with SRB. These variables were selected a priori based on previous literature demonstrating their association with SRB variation and differential vulnerability to population-level stressors.

Mothers were grouped by their COB rather than region of birth, as significant differences in reproductive health practices [31] and the impact of the pandemic [28] exist among mothers from the same region. Additionally, preferences for male or female offspring may differ between countries within the same region. Maternal COB was modelled as a categorical variable. Due to sample size considerations, the three largest groups—non-Indigenous Australian, Indian, and Chinese—were analysed separately, while all other COB were combined into a single “Other” category. This category comprised mothers from multiple countries, each contributing relatively small numbers of births, and was included to preserve model stability and interpretability.

Socio-Economic Indexes for Areas (SEIFA) were assigned using the maternal Local Government Area (LGA) of residence (ABS SEIFA deciles; 1 = most disadvantaged, 10 = most advantaged) [33]. For analysis, SEIFA deciles were collapsed into four categories to ensure adequate numbers within each stratum: most disadvantaged (deciles 1–3), 2nd most disadvantaged (deciles 4–5), 2nd most advantaged (deciles 6–7), and most advantaged (deciles 8–10).

### Data analyses

Descriptive statistics were used to summarise the number and sex of babies born each month from 2016 to 2023. The SRB was calculated as the number of male births per total number of births. The Chi-square test and Student’s t-test were used to assess associations between categorical variables and continuous variables, respectively. A significance level of *p* < 0.05 was considered statistically significant.

Multivariable logistic regression was used to identify factors associated with SRB. The periods June-August and December 2020 were compared to other time periods. In addition to these time periods, demographic characteristics, maternal health, and reproductive history were included in the full models. An interaction between age and number of pregnancies was identified, but the interaction term was not significant and was thus removed from the final models. Logistic regression was also conducted separately for each of the three largest maternal COBs.

Time series analysis was conducted using an ARIMA model, with data from January 2016 to March 2020 representing the pre-pandemic period. Monthly SRB values (male live births divided by total live births per month) were used as the dependent variable. Deviations during the pandemic period were identified when observed SRB values fell outside the 95% confidence interval of predicted pre-pandemic values. The model was then applied to forecast the SRB for the pandemic period, extending through to December 2024. Change in the SRB during the pandemic compared to the pre-pandemic period was determined by identifying outliers falling outside the 95% confidence interval (CI) of the predicted values. We used the autoArima() function in R to perform a grid search of multiple models and selected the model with the lowest Akaike Information Criterion (AIC) value. The AIC was used to evaluate models by balancing goodness of fit with model simplicity, thereby avoiding overfitting while ensuring an optimal fit. This approach allowed for the identification of the most appropriate model for each cohort, considering both seasonal and non-seasonal components. Separate models were generated for the full cohort and the three main maternal COB: non-Indigenous Australian, Indian and Chinese, with the selected models outlined in Appendix 1. To validate model fit, we examined the autocorrelation function (ACF) and partial autocorrelation function (PACF) plots, which supported the identified models and showed no additional periodic patterns. Data were analysed using Stata 18 [34] and R v.4.3.1. Ethics approval was obtained from the Local Health District Human Research Ethics Committee (2025/PID00316–2025/ETH00271).

## Results

A total of 119,756 singleton births occurred for residents of WSLHD between 2016 and 2023. On average, there were 1,248 births per month, or 14,970 births per year. February had the fewest births, with an average of 1,174 births, while March had the highest, with an average of 1,304 births (*p* < 0.001).

### Maternal characteristics

More than one-third of mothers were aged 30–34 years (38.7%). The three largest COBs were non-Indigenous Australian (37.2%), India (16.2%) and China (7.4%). The majority of mothers resided in either the most dis-advantaged areas (25.8%) or the second-most disadvantaged areas (38.5%). Approximately one-quarter of the mothers were overweight (27.2%) and one-fifth were obese (19.1%). A small proportion had pre-existing conditions, such as diabetes (1.2%), hypertension (1.1%), or hepatitis B (0.8%). Mothers with no previous pregnancies made up the largest proportion (41.6%), followed by those with one previous pregnancy (36.2%) (Table 1).

### Pandemic associations with SRB

**Fig. 1:**
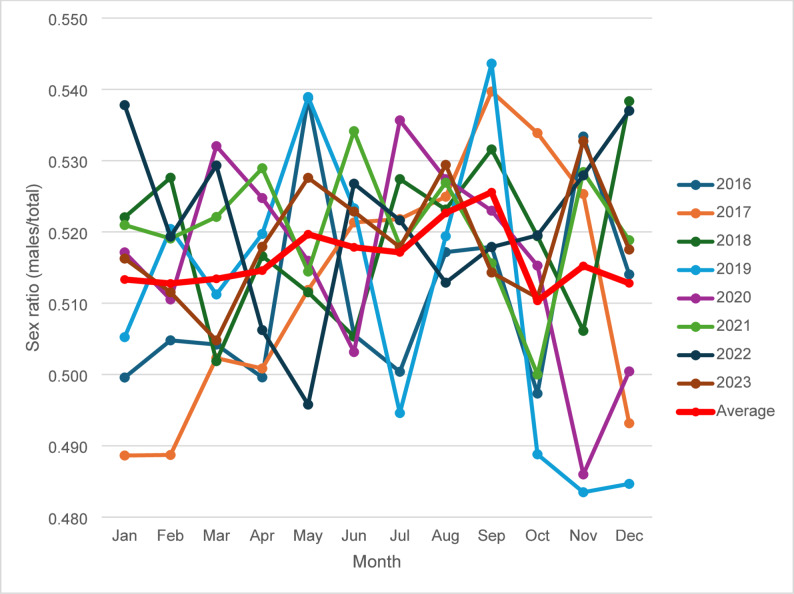
Sex ratio (males/total births) by month in Western Sydney Local Health District, 2016–2023

The total number of male births was 61,832, yielding an SRB of 0.516, or 106.7 males per 100 females. The SRB was slightly higher in September 2020 (average of 0.526) and lower in October 2020 (average of 0.510) (Fig. 1).

The SRB of 0.522 during the June–August 2020 period was not statistically different to the SRB of 0.516 during other periods (Adjusted odds ratio [aOR] = 1.032; 95% CI: 0.925–1.151). Similarly, the SRB of 0.500 during December 2020 was not statistically significant (aOR = 0.939; 95% CI: 0.836–1.055) (Table 1). However, when analysed by COB, Chinese mothers giving birth between June and August 2020 had higher odds of having a male baby compared to other periods (SRB = 0.621 vs. 0.521; aOR = 1.557; 95% CI: 1.194–2.030). During these months, the average monthly number of female births was notably reduced, from 44.0 to 30.1, while the average monthly number of male births increased slightly, from 47.9 to 50.3 (Table 2).

**Table 2:** Logistic regression models of factors associated with sex ratio at birth by maternal country of birth in WSLHD between 2016 and 2023

	Australia, non-Indigenous			India			China		
	aOR	95% CI		aOR	95% CI		aOR	95% CI	
TIME									
June-August 2020	1.032	0.925	1.151	0.999	0.855	1.168	1.557**	1.194	2.030
December 2020	1.099	0.904	1.336	0.848	0.649	1.108	0.961	0.589	1.568
Other times	Ref.			Ref.			Ref.		
AGE (years)									
14–24	1.030	0.968	1.096	1.063	0.895	1.262	0.774	0.599	1.001
25–29	1.030	0.982	1.080	0.976	0.907	1.050	1.043	0.932	1.166
30–34	Ref.			Ref.			Ref.		
35–39	0.965	0.912	1.020	1.034	0.959	1.115	1.055	0.944	1.179
>=40	0.898*	0.809	0.997	1.024	0.833	1.257	0.985	0.784	1.237
SEIFA INDEX									
Most disadvantaged	Ref.			Ref.			Ref.		
2nd most disadvantaged	1.048	0.986	1.114	1.140*	1.024	1.271	1.038	0.885	1.219
2nd most advantaged	1.055	0.998	1.115	1.083	0.974	1.206	0.857	0.740	0.992
Most advantaged	0.997	0.946	1.050	1.080	0.967	1.206	0.908	0.798	1.034
HEIGHT									
Shortest decile	1.050	0.954	1.156	0.988	0.820	1.191	0.959	0.728	1.264
Medium (2nd-9th deciles)	Ref.			Ref.			Ref.		
Tallest decile	0.993	0.943	1.046	1.007	0.846	1.198	1.003	0.826	1.218
BODY MASS INDEX									
Underweight (< 18.5)	0.882*	0.788	0.987	1.121	0.946	1.328	1.054	0.924	1.202
Healthy weight (18.5-<25)	Ref.			Ref.			Ref.		
Overweight (25-<30)	0.977	0.933	1.023	0.987	0.926	1.052	0.997	0.875	1.136
Obese ( > = 30)	1.014	0.967	1.064	1.028	0.939	1.125	0.922	0.697	1.221
PRE-EXISTING DIABETES									
No	Ref.			Ref.			Ref.		
Yes	0.940	0.775	1.139	1.004	0.784	1.286	1.338	0.739	2.425
HYPERTENSION									
No	Ref.			Ref.			Ref.		
Yes	0.981	0.822	1.172	1.107	0.817	1.500	0.740	0.362	1.509
HEPATITIS B									
No	Ref.			Ref.			Ref.		
Yes	0.858*	0.755	0.976	0.980	0.555	1.729	1.024	0.836	1.255
PREVIOUS PREGNANCY									
0	Ref.			Ref.			Ref.		
1	1.031*	1.004	1.059	1.079*	1.013	1.149	1.062	0.966	1.166
2	1.050	1.012	1.090	1.238**	1.081	1.418	1.062	0.903	1.249
>=3	1.062	0.990	1.139	1.066	0.788	1.442	1.023	0.739	1.417

*p < 0.05; **p < 0.01; ***p < 0.001

The results from the logistic regressions were supported by results from the timeseries analyses. The ARIMA analysis revealed distinct patterns in the sex ratio across cohorts during the pandemic period. In the full cohort, SRB values in March 2022, May 2022 and August 2022 exceeded the upper 95% confidence limits. Among Indian mothers, deviations occurred in April 2022, June 2022 and September 2022. These findings indicate a delayed, sustained increase beginning in 2021 rather than an immediate post-pandemic effect.

**Fig. 2:**
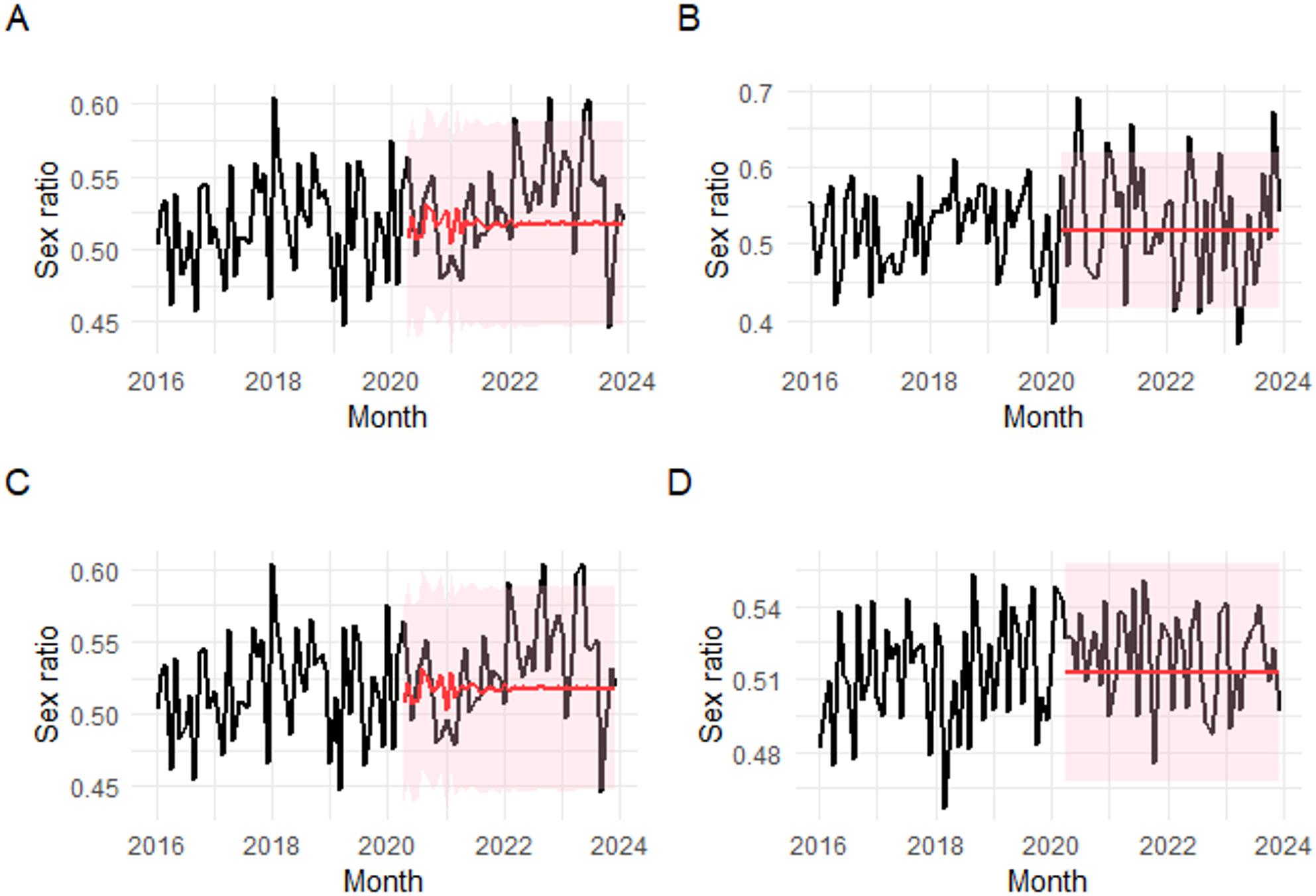
Time series analyses of sex ratio at birth in WSLHD between 2016 and 2023. **A** All women, **B** China, **C** India, **D** Non-Indigenous Australia. Black line: Actual; Red line: Predicted

In contrast, the Chinese subgroup exhibited high fluctuations, with four months (July 2020, June 2021, June 2022 and November 2023) surpassing the upper confidence limit and three months (March 2022, August 2022 and April 2023) falling below the lower limit. The non-Indigenous Australian subgroup showed no significant deviations, with sex ratio values remaining within the expected range. These findings suggested pandemic-associated shifts in the sex ratio, particularly among the Indian and Chinese cohorts, alongside a broader increase observed in the overall population (Fig. 2 and Appendix 1).

### Factors associated with SRB

In the multivariable logistic regression model in Table 1, mothers from India had a significantly higher SRB than non-Indigenous Australian mothers (SRB = 0.524 vs. 0.516; aOR = 1.038; 95% CI: 1.001–1.076). Compared to mothers with no previous pregnancies, those with one previous pregnancy had a higher SRB (SRB = 0.519 vs. 0.513; aOR = 1.031; 95% CI: 1.004–1.059), as did mothers with two previous pregnancies (SRB = 0.522 vs. 0.513; aOR = 1.050; 95% CI: 1.012–1.090).

Conversely, mothers with hepatitis B had a lower SRB compared to those without (SRB = 0.483 vs. 0.517; aOR = 0.858; 95% CI: 0.755–0.976). A total of 977 mothers (0.8% of the total sample) were positive for hepatitis B (Table 1). The majority of these mothers were from China (434 or 44.4% of all hepatitis B-positive mothers). However, no significant difference in SRB was observed between Chinese hepatitis B-positive and negative mothers (p from chi-squared test = 0.447).

Chinese mothers residing in the second-most advantaged areas had lower odds of giving birth to a male baby compared to those living in the second-most disadvantaged areas (SRB = 0.510 vs. 0.544; aOR = 0.857; 95% CI: 0.740–0.992).

## Discussion

This study examined temporal changes in the SRB during the COVID-19 pandemic in a large multicultural population. We found no immediate aggregate shift in SRB during the early pandemic period. However, subgroup-specific variation by maternal country of birth (COB) was evident, particularly a short-term increase among Chinese-born mothers and persistently elevated SRB among Indian-born mothers.

The absence of an overall population-level change was consistent with findings from the United States [11] but contrasts with findings from England and Wales [1] and from Ireland [24]. The relatively modest public health impact of the pandemic in Australia [35, 36] may partly explain the absence of an overall population-level shift in SRB.

Although no immediate aggregate change was detected, time-series analyses suggested a delayed increase in SRB beginning in 2021. This pattern differs from the acute declines in male births reported following sudden disasters and may reflect prolonged, lower-intensity stressors during later pandemic stages, such as sustained economic uncertainty and ongoing changes in healthcare access, rather than acute stress-related foetal loss mechanisms. However, individual-level measures of psychological stress and behavioural change were not available, and these interpretations should therefore be considered exploratory.

A short-term increase in SRB was observed among births to Chinese-born mothers during the three-to-five-month period following pandemic onset. Migrant populations may experience distinct stressors during public health emergencies, including social isolation, employment instability, disrupted access to healthcare, and concerns for family members overseas, potentially amplified by transnational information exposure. These factors may interact with pre-existing social or cultural determinants of reproductive behaviour following migration, although the available data do not permit direct examination of these pathways. Sex-selective abortion has been documented in some countries and populations with a cultural background of son preference and has been discussed as a contributor to elevated SRB [17, 18, 37, 38]. However, this study could not assess the contribution of sex-selective practices in Australia due to the absence of termination data. Notably, the timing of the observed increase corresponds to early gestation, when stress-related biological mechanisms affecting foetal viability have been proposed [20–23]. Behavioural mechanisms such as termination are less likely to fully account for this pattern. However, behavioural explanations cannot be excluded, and causal inferences cannot be drawn from the available data.

Interestingly, Chinese mothers living in more advantaged areas were less likely to have a male infant. This suggests that area-level socio-economic status may operate differently across cultural contexts and that material advantage alone does not uniformly predict SRB patterns.

Indian-born mothers exhibited the highest overall SRB across the study period, independent of pandemic timing. India is known for its cultural preference for boys over girls, with a reported ratio of 108 boys for every 100 girls between 2016 and 2023, exceeding the global average of 105 [39]. While sex-selective abortion has been a significant factor influencing SRB in India [37, 38], their relevance among Indian migrant populations in Australia cannot be determined in this study. Nevertheless, the persistence of higher SRB across multiple years suggests structural or cultural influences beyond pandemic-related effects.

Among non-Indigenous Australian mothers, older mothers were less likely to have a male baby. This is consistent with the findings of a study conducted in the United States [11]. Older mothers may experience higher levels of stress during pregnancy due to factors such as aging reproductive systems, increased prenatal depression, elevated cortisol levels [40], and higher rates of chronic health conditions [41]. Similar to finding from previous research [14], non-Indigenous Australian mothers who were underweight were less likely to have a male baby. The Trivers-Willard hypothesis suggests that mothers in poor physical conditions are more likely to give birth to female offspring to maximize reproductive success because female offspring are more likely to survive infancy and childhood than male offspring [42, 43].

Hepatitis B infection was also associated with a lower likelihood of having a boy among non-Indigenous Australian mothers. However, the number of non-Indigenous Australian mothers with hepatitis B was relatively small (49). More research on this population is needed before drawing any firm conclusions. In contrast, a substantial number of Chinese mothers (434) had hepatitis B, but no association with SRB was detected. The relationship between hepatitis B and SRB among Chinese mothers has been inconsistent in previous studies. Older studies suggested a positive relationship [44], while more recent studies have shown either a weak association [16] or no association at all [45]. It is possible that the effect of hepatitis B on SRB varies depending on the population.

Overall, the findings indicate that SRB patterns during the COVID-19 pandemic were not uniform across maternal groups and were shaped by a combination of biological, reproductive, and social factors. While this study does not support an immediate population-level association between the pandemic and SRB, the observed heterogeneity by maternal country of birth highlights the importance of examining subgroup-specific patterns in culturally diverse populations.

### Strengths and limitations

This is the first study on SRB by COB in Australia. It included a large sample size of nearly 120,000 babies, allowing for the detection of small changes in SRB and the effects of covariates. The study spanned a period of 48 months before and 48 months after the start of the pandemic, enhancing the accuracy and relevance of the comparison. The use of both multivariable logistic regression and ARIMA time-series modelling provided complementary analytical perspectives, enabling assessment of both short-term deviations and longer-term temporal trends. We included births from mothers of different COBs to reflect the population of the WSLHD, as well as from the largest COBs to highlight differences in associated factors. The results could be applicable to similar settings.

The limitations of this study include the lack of data on paternal COB. Intermarriage is common in Australia, with 31.6% of all marriages in 2016 involving mixed-culture couples [46]. Paternal culture may strongly influence son preference and impact couples’ decisions. Other potential factors such as parental education, occupation, and income were also not available. In addition, data on pregnancy terminations, including foetal sex prior to viability, were not available, precluding assessment of the potential contribution of sex-selective practices to observed SRB patterns. Future studies should consider including these factors wherever possible. Since the study was conducted at the population level, it was not possible to understand the mechanisms by which these factors influence SRB at the individual level.

This study is observational and based on routinely collected administrative data. As such, causal inference regarding the impact of the pandemic cannot be made. Individual-level measures of psychological stress, reproductive intention, healthcare access, sexual behaviour, and termination data were not available. Residual confounding from unmeasured factors cannot be excluded.

## Conclusions

The COVID-19 pandemic was not associated with an immediate overall change in SRB in Western Sydney. However, heterogeneous patterns by maternal country of birth were observed, including a short-term increase among Chinese mothers and persistently higher SRB among Indian mothers.

These findings suggest that strategies to address SRB imbalances should be tailored to the contexts of specific maternal populations. Reproductive health responses during public health crises should incorporate culturally informed engagement, equitable access to services, and community-based strategies that promote gender equity and reproductive autonomy. Strengthening surveillance systems to include termination data and key social determinants would improve understanding of the mechanisms underlying SRB variation.

In conclusion, a proactive, culturally responsive, and equity-focused reproductive health framework is essential to ensure that SRBs remain balanced and aligned with human rights principles in increasingly diverse populations.

## Supplementary Information

Supplementary Material 1.

## Data Availability

No datasets were generated or analysed during the current study.
