# Supplementary material for: Birth sex ratio in Western Sydney during the COVID-19 pandemic: associations by maternal country of birth
Source: Reprod Health. 2026 Apr 24;23:111. doi: 10.1186/s12978-026-02304-1 (PMC13248341; doi:10.1186/s12978-026-02304-1)
Supplement: Supplementary file 1 — Supplementary Material 1. [file 12978_2026_2304_MOESM1_ESM.docx]

**Appendix 1. Time series analysis using ARIMA model of changes in sex ratio at birth in WSLHD between 2016 and 2023 by country of birth**

| Cohort | Fitted ARIMA model | Model description | Model coefficient | | Model diagnostics | |
| --- | --- | --- | --- | --- | --- | --- |
|  |  |  | Seasonal autoregression | Mean | Sigma^2^ | AIC |
| Full cohort | (0,0,0)(1,0,0)[12] | Seasonal autoregressive model – with a period of 12 months | -0.242 | 0.517 | 0.001 | -193.18 |
| Australia | (0,0,0) | White noise model | - | -0.512 | 0.001 | -237.17 |
| China | (0,0,0) | White noise model | - | 0.517 | 0.002 | -153.86 |
| India | (0,0,0)(1,0,0)[12] | Seasonal autoregressive model – with a period of 12 months | -0.244 | 0.517 | 0.001 | -192.89 |
